# A Model of Non-Homologous Recombination Mediated by HIV-1 Reverse Transcriptase Explaining Sequence Motif Duplications That Confer a Replication Fitness Advantage

**DOI:** 10.3390/v17050680

**Published:** 2025-05-07

**Authors:** Arun Panchapakesan, Udaykumar Ranga

**Affiliations:** 1 HIV-AIDS Laboratory, Molecular Biology and Genetics Unit, Jawaharlal Nehru Centre for Advanced Scientific Research, Jakkur, Bengaluru 560064, India; arun@yrgcare.org; 2 Molecular Biology Laboratory, Y R Gaitonde Centre for AIDS Research and Education (YRG CARE), Chennai 600031, India

**Keywords:** HIV, reverse transcriptase, sequence duplication, non-homologous recombination

## Abstract

The Reverse Transcriptase of the Human Immunodeficiency Virus (HIV) is distinguished by its high rate of homologous recombination. A less-studied consequence of this phenomenon is the increased occurrence of non-homologous recombination, which results in length polymorphism. While most of these genome-wide variations are sporadic, some provide a replication advantage to variant strains, such as those in the Long Terminal Repeat (LTR) and p6-Gag regions. By analyzing sequences from these two regions in the HIV-1 databases, we categorize all types of non-homologous recombination into four groups based on the presence or absence of two molecular features. Additionally, drawing on established models of homologous recombination, we propose a model that describes the process of sequence duplication. This model can also be applied to explain non-homologous recombination in different types of HIV and other viruses.

## 1. Introduction

The Reverse Transcriptase (RT) of the Human Immunodeficiency Virus is notable for its ability to generate variants at a remarkably high rate. This ability is primarily driven by three molecular features—first, the low fidelity of the RT, which is due to the lack of proofreading activity [1,2]; second, the pseudo-diploid nature of the viral genome, allowing the enzyme to switch between two viral RNA strands to generate recombinants; and third, the rapid rate of replication of the virus, enabling the production of multiple variant strains [2,3]. These unique qualities collectively enable the virus to exist as a highly variable quasispecies within the host. As a result, over 95% of viral genomes in a host are defective due to large internal deletions, hypermutations (catalyzed by the antiviral activity of the APOBEC family of enzymes [3,4]), premature stop codons, and/or frameshift mutations.

While significant focus has been placed on genetic variations of different types within the viral genome, relatively limited attention has been given to sequence duplications, which are present in approximately 17% of HIV-1B and HIV-1C sequences in the Los Alamos Sequence database. Sequence duplications, though mainly sporadic, can occur at several locations of the viral genome. These variant viral strains are often overlooked due to the sporadic nature of the duplications, their relatively low frequency, and the absence of apparent positive selection and expansion [5,6,7]. For instance, sequence duplications have been reported at the Sp1 sites in the Long Terminal Repeat (LTR [5,8]), Trans-Activation Response (TAR) element [9], RT [10,11], *env* [12], and *nef* genes [13]. However, evaluating the biological significance of a sequence motif duplication can be challenging when only a few additional amino acid residues are added to the reading frame of a viral protein without causing a frameshift mutation. For example, the significance of adding only three additional amino acid residues to the PTAP motif of the p6-Gag of HIV-1B is not well understood, although this modification represents the most common sequence duplication in this subtype [14]. Approximately 4.7% of HIV-1B sequences in the databases contain the partial duplication of the PTAP motif [14].

Against this backdrop, we highlight a class of sequence motif duplications in the HIV-1 genome that occur at a much higher frequency in natural infection and, importantly, appear to confer a significant replication advantage on the variant viral strains. In HIV-1C, we identified and characterized two such hotspots of sequence motif duplications in the LTR and p6-Gag. For example, variant viral strains harboring an additional copy of the PTAP motif in p6-Gag dominate the canonical viral strains in both the plasma and the latent viral reservoir compartments [14]. Similarly, we previously demonstrated that the presence of an additional copy of the NF-κB motif in the HIV-1 LTR enhances transcriptional strength, allowing the variant viral strains to dominate the canonical strain in several experimental models and, importantly, in natural infection [15].

Of note, we recently demonstrated the emergence of several LTR-variant viral strains in HIV-1C that contain additional copies of existing transcription factor binding sites (TFBSs) created by sequence duplication [16]. These viral strains are characterized by variations in the TFBS copy number, sequences of the duplicated motifs, and the relative positions of the duplicated motifs. The overall profile of TFBSs in these viral LTRs appears to influence the transcriptional activity and latent reservoir properties of the virus. Of particular importance are the duplications in the modulator–enhancer junction of the HIV-1C LTR, where two critical TF binding sites, NF-κB and RBEIII, play opposing roles in viral transcription [15,17]. NF-κB generally enhances transcription in activated cells, while RBEIII contributes to repression in resting cells [17]. Sequence duplications involving these motifs are common and profoundly impact LTR function [15,16,17]. The duplication of the NF-κB motif increases transcriptional activity and enhances reactivation from latency, whereas the duplication of the RBEIII motifs reinforces transcriptional silencing. Importantly, the balance between activating and repressing motifs appears to determine the transcriptional output of the LTR. For example, increasing the copies of the NF-κB motif while keeping that of the RBEIII motif constant progressively enhances reactivation. In contrast, reducing NF-κB copies leads to transcriptional quiescence (Bhange D et al., unpublished data). These results suggest that LTR architecture finetunes the depth and stability of latency in HIV-1C, with natural variation in the TFBS copy number contributing to differences in viral persistence and responsiveness to therapeutic reactivation. Thus, the events of TFBS duplication within the LTR do not merely amplify TF binding but also reshape the regulatory circuit that governs viral latency.

A careful observation of sequence motif duplication in these two hotspots of the HIV-1 genome, p6-Gag and the LTR, confirms that the phenomenon is highly diverse despite a common underlying framework, indicating potentially diverse mechanisms contributing to the duplication of each motif independently. Although sequence duplications at these two hotspots are shared among all HIV-1 subtypes, subtype-specific traits are evident, especially in HIV-1C. For example, NF-κB motif duplication in the viral enhancer, which can enhance the transcriptional strength of the LTR, is unique for HIV-1C and not seen in other HIV-1 genetic families [15]. The creation of the fourth copy of the NF-κB motif in HIV-1C LTR is genetically distinct yet highly conserved, regardless of geographical and temporal differences.

The canonical HIV-1C LTR contains three copies of the NF-κB motif, two of an identical sequence motif (5′-GGGACTTTCC-3′, labeled the H-κB site) and one with a sequence variation (5′-GGGGAGTTTCC-3′, called the C-κB site, differences underlined). Given the order of these three NF-κB motifs from the 5′ end to the 3′ end, the viral promoter is designated as HHC-LTR. In a variant viral strain containing four copies of the NF-κB motif (designated FHHC, ‘F’ representing the fourth motif, 5′-GGGACTTTCT-3′), a sequence of 22 base pairs (5′-GCTGACACAGAAGGGACTTTCT-3′), comprising the upstream H-κB motif and the sequences immediately upstream, is copied. Notably, the ultimate base at position 10 of the F-κB motif is invariably changed to a ‘T’, thus distinguishing the F-κB motif from the canonical H-κB motif through a ‘C-to-T’ substitution at position 10.

In contrast to the highly faithful duplication of the F-κB motif of precisely 22 base pairs, the duplication of the RBEIII motif (designated as ‘R’) in the modulatory region of the LTR exhibits significant length, ranging from 10 to 33 residues and genetic variations in the inserted sequences [16]. Further, while the RBEIII motif core sequence of eight bases (5′-ACTGCTGA-3′) is fully conserved following sequence duplication, the co-duplicated flanking sequence motifs, which serve as binding sites for other transcription factors, harbor subtype-specific variations possibly modulating latency kinetics among different HIV-1 subtypes. Furthermore, sequence motif duplication in HIV-1C becomes more complicated with permutations and combinations of several TFBSs, even blurring the distinction between the enhancer and modulatory regions of the viral promoter and leading to the generation of several LTR-variant viral strains.

A similar degree of sequence length variation is evident among HIV-1 subtypes in the duplication of the PTAP motif in p6-Gag. In HIV-1B, only a partial PTAP motif is duplicated, the biological significance of which is not fully established, although sporadic reports implicate a compensatory role in drug resistance [18]. In contrast, in HIV-1C, not only is the motif duplicated in its entirety at a significantly higher frequency, but the length of the duplicated motif is also highly variable, ranging from three to fourteen amino acids, although the core motif sequence (PTAPP) is highly conserved [14,19].

Thus, given the complex nature of sequence motif duplications in HIV-1C, comprising copy number, sequence, combinatorial, and flanking sequence variations, a single molecular model cannot satisfactorily explain the phenomenon, warranting a broader classification schematic. RT-mediated recombination during reverse transcription between the two viral genome copies co-packaged into the viral particles is the basis for sequence motif duplications in the viral genome. Sequence duplications arise due to non-homologous, rather than homologous, recombination events mediated by HIV-1 RT.

Several studies have characterized homologous recombination in HIV-1, which is estimated to occur at a frequency of three to five template switches between the two copies of the viral genome per round of viral replication [1]. In homologous recombination, an absolute or significant complementarity must exist between the nascent DNA and the template RNA. The most widely accepted model for homologous recombination in retroviruses is the dynamic copy-choice model [20]. This model posits that a template switch is primarily driven by the disruption of the delicate equilibrium between the polymerase and RNase H activities of RT. Variations affecting either of these enzyme activities or the presence of RNA secondary structures can influence the rate of recombination.

A related model, the dock-and-lock model, suggests that acceptor strand invasion catalyzes the hybridization of the nascent DNA to the acceptor RNA, slowing the RT and thus facilitating strand switching [21,22,23]. In this model, the acceptor RNA progressively hybridizes with the nascent complementary DNA (cDNA), eventually displacing the donor RNA molecule and prompting the enzyme to switch to the acceptor RNA-cDNA hybrid for continued polymerization [24,25,26].

In contrast, non-homologous recombination, essential for sequence motif duplication, is expected to operate in the absence of perfect sequence complementarity, is much rarer, and has been less frequently studied. The frequency of non-homologous recombination is estimated to be at least a thousand times lower than that of homologous recombination [27]. Therefore, while the dynamic copy-choice paradigm and dock-and-lock model offer insights into viral recombination in HIV-1, a more comprehensive framework is needed to address the complexities associated with sequence motif duplications.

Here, using data from existing sequence databases and our own observations, we provide a scaffold for classifying sequence motif duplications into four distinct subgroups. Additionally, we propose a model to explain the mechanism by which HIV-1 RT generates these sequence duplications. Given that duplications are more frequent and varied in HIV-1C [14,15,16], we will use this subtype as the point of reference while delineating the salient features of the phenomenon.

## 2. A Classification of Sequence Motif Duplications in HIV-1

Two distinct molecular traits—the presence of a triplet motif flanking the sequence to copy and the presence of a mismatched base pair at the 3′ end of the nascent DNA—permit the classification of the sequence motif duplications in the HIV-1 genome into four groups as outlined below. The triplet motif flanking the duplicated motif lacks a defined label. It has been referred to variously as a short direct repeat [28], short regions of homology [29], or short regions of sequence identity [30]. A search of HIV-1 sequences in the LANL database identified sequences with or without flanking repeat sequence motifs (see below). Furthermore, we observed the presence of a mismatched base at the 3′ terminal end following a strand switch (see Section 2.2 and Section 2.4), leading to the creation of additional variation in the duplicated sequence.

**Figure 1:**
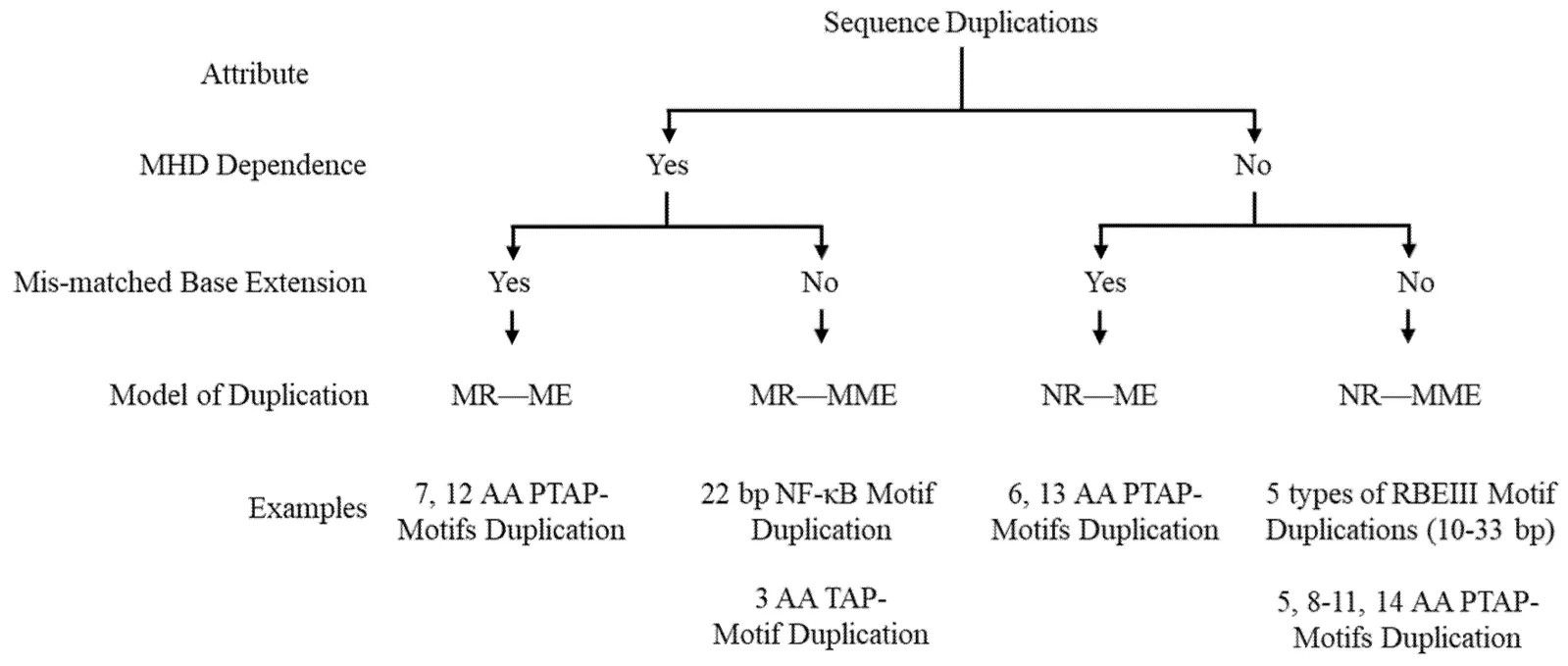
Classification of Sequence Duplications. A schematic depiction of a classification system categorizing non-homologous recombination events in HIV-1 leading to sequence motif duplication of four types. MR—ME: MHD-dependent recombination and matched-base extension. MR—MME: MHD-dependent recombination and mismatched-base extension. NR—ME: Non-MHD-dependent recombination and matched-base extension. NR—MME: Non-MHD-dependent recombination and mismatched-base extension.

Based on these two molecular features, the presence or absence of the triplet motif flanking the duplicated sequence and the presence or absence of a mismatched base at the growing end of the nascent DNA following strand switch, we classify the non-homologous recombination of HIV-1 into four categories (Figure 1). Importantly, we suggest a label for the three-base motif, the ‘micro-homology domain’ (MHD), since such a sequence of only three bases or even smaller can permit non-homologous recombination (see Section 3). The proposed classification system is further corroborated by the observation that these sequence duplications occur at an increased frequency when either of these molecular features, i.e., the MHD or a matched terminal base pair (Figure 1), are present in the duplicated sequence [14,15]. Further, the four models of recombination we describe here can explain every kind of sequence motif duplication identified in the two hotspots of the HIV-1 genome and possibly elsewhere. These models, especially the MR—ME model, can be applied to explain sequence motif duplications observed in other viruses, including Influenza [31], SARS-Cov-2 [32], Hepatitis E [33], and the Japanese Encephalitis Virus [34]. The relatively low recombination rates mediated by the RNA polymerases in these viruses make the frequency of these events rarer compared to that in HIV-1 [1].

Since these sequence duplications appear at the highest frequency in HIV-1C, we will use this subtype as a reference in our classification. Additionally, while naming the variant viral strains, we will follow the nomenclature established previously [17,18,19].

### 2.1. The MR—ME Duplication

**Figure 2:**
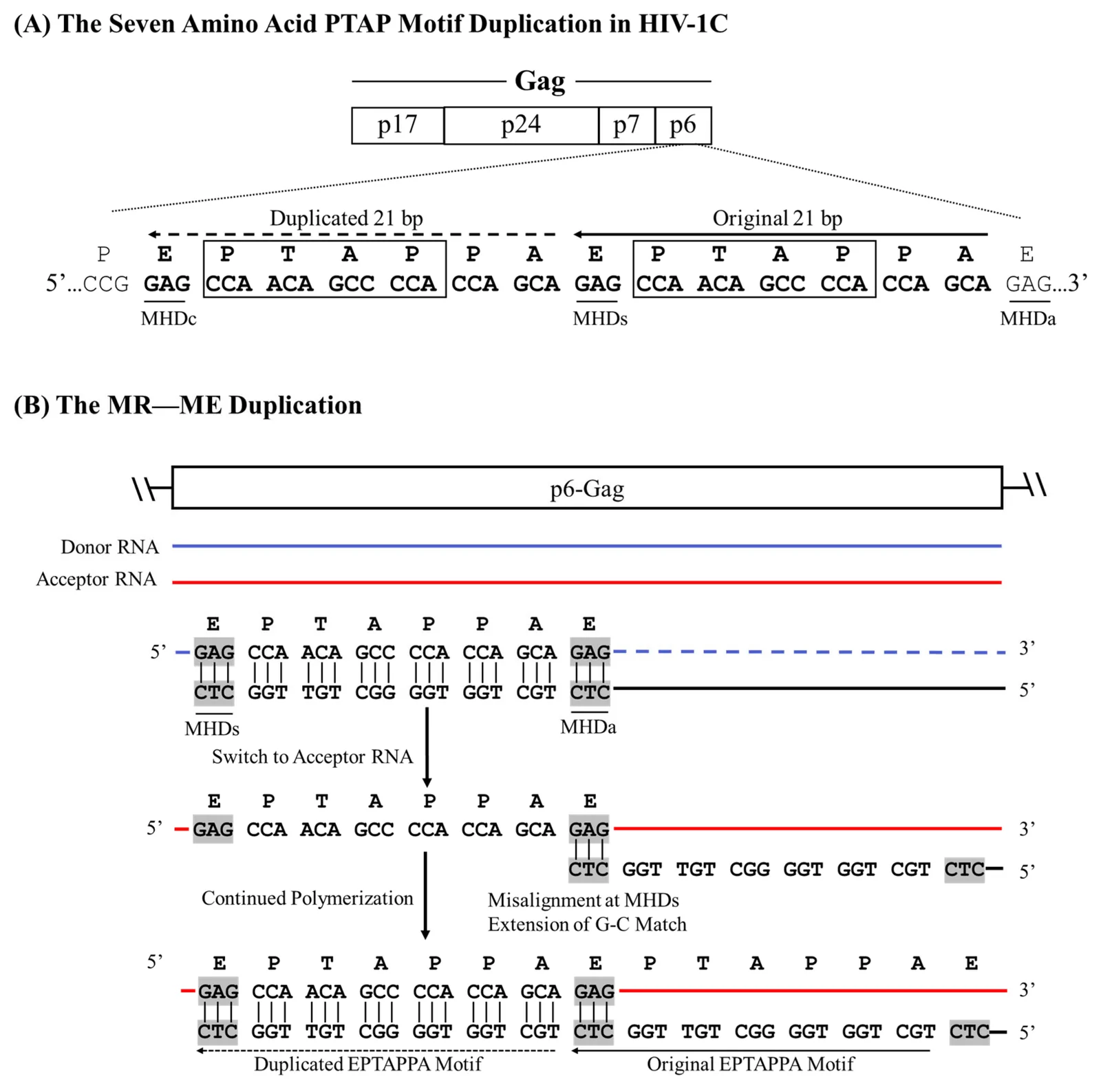
A schematic portraying the seven amino acid PTAP motif duplication in HIV-1C by the MR—ME mechanism. (**A**) Two copies of the PTAP motif of seven amino acid length (EPTAPPA) and the corresponding nucleic acid sequence are presented. Solid and dashed arrows represent the original and the duplicated sequences, respectively. The MHDs, MHDa, and MHDc triplets are underlined. The 21-base sequence in p6-Gag corresponds to 2145–2169 coordinates in HXB2. (**B**) The donor and acceptor RNAs are shown using blue and red lines, respectively, and the black line represents the nascent cDNA. Dashed lines represent the template RNA degraded by the RNase H activity of the RT. The MHD motifs are highlighted in gray.

This type of duplication occurs when a perfectly conserved triplet MHD flanks the sequence to be duplicated and the base present at the 3′ end on the cDNA is complementary to the base on the acceptor RNA. PTAP motif duplications of three different lengths (7, 12, and 14 aa) can be classified into this category (Figure 1). For illustration, we consider the example of the seven amino acid core PTAP motif (EPTAPPA) duplication of HIV-1C [14]. A 21-base pair sequence (5-GAG CCA ACA GCC CCA CCA GAA-3′) encoding the core PTAP motif is duplicated and inserted immediately downstream of the original motif as the RT continues to polymerize. Analysis of the nucleic acid sequence identifies a unique triplet, ‘GAG’, flanking both motifs. When a single PTAP motif is encoded by the RNA sequence, two copies of the GAG triplet are present at either end of the sequence to be duplicated. In contrast, three copies of the GAG triplet are found flanking the two copies of the PTAP motif core sequence following duplication (Figure 2A), suggesting that the GAG triplet functions as the MHD for the PTAP sequence duplication of 21 base pairs. The three MHD motifs are designated as MHDs for the motif ‘stalling’ RT polymerization, MHDa for the motif ‘accepting’ the cDNA at template switching, and MHDc for the motif created as an additional copy.

Based on this observation, we propose the following schema for the seven-amino-acid PTAP motif duplication. The RT stalls after copying the 5′ G-residue of the GAG triplet (the MHDs motif) on the donor RNA, prompting the nascent cDNA to switch strands from the donor RNA to the acceptor RNA molecule. When the RT switch is faithfully aligned with the complementary sequence on the acceptor RNA, which is expected to be the most common event, polymerization will resume without sequence motif duplication. However, in a rare event, the ‘CTC’ triplet present at the 3′ end of the cDNA may misalign with the GAG triplet (the MHDa motif) present at the 3′ end of the sequence to be copied on the acceptor RNA template (Figure 2B). Notably, there is no mismatch at the growing end of the cDNA in this scenario as the ‘CTC’ triplet of the cDNA is a perfect match to the ‘GAG’ triplet on the acceptor RNA molecule. As the RT resumes polymerization, from these misaligned complementary sequences, the 21 bases encoding the seven amino acid sequences of the PTAP motif are copied again, creating an additional copy of the PTAP motif, including the GAG triplet (the MHDc motif). We designate this class of duplications as MHD-dependent recombination and matched-base extension (MR—MR—ME).

### 2.2. The MR—MME Duplication

**Figure 3:**
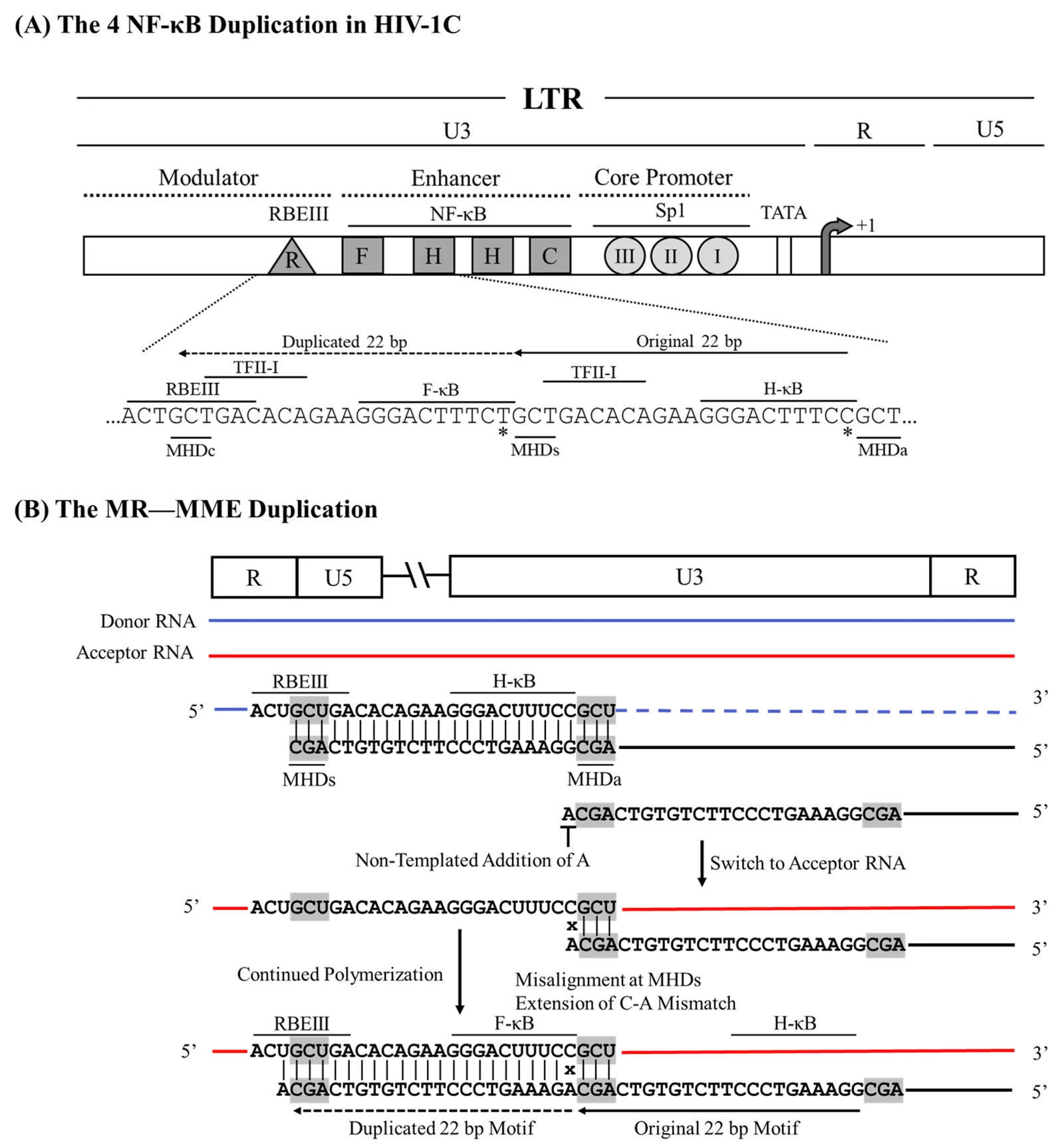
A schematic depicting the 22 bp NF-κB motif duplication in HIV-1C by the MR—MME model. (**A**) The architecture of the HIV-1C LTR depicting the relative positions of the four NF-κB and other important TFBSs, along with their nucleic acid sequences. Solid and dashed arrows represent the original and the duplicated sequences, respectively. MHDs, MHDa, and MHDc triplets are underlined. The asterisks represent the C-to-T variation between the H- and F-κB motifs. The 22-base pair sequence in the LTR corresponds to 325–352 coordinates in HXB2. (**B**) The donor and acceptor RNAs are shown using blue and red lines, respectively, and the black line represents the nascent cDNA. Dashed lines represent the template RNA degraded by the RNase H activity of the RT. The mismatched base that is extended is represented with an x. The MHD motifs are highlighted in gray.

The creation of a fourth copy of the NF-κB motif in the HIV-1C LTR, which confers a significant replication fitness advantage on the variant viral strains [15], serves as an excellent example when the RT uses the MHD-dependent recombination strategy leading to sequence motif duplication. However, unlike in the MR—MR—ME type of duplication described above, the RT here encounters a mismatched base at the growing end of the cDNA that is non-complementary to the acceptor RNA template. The new κB-motif added to the viral enhancer, called the F-κB motif, demonstrates distinct molecular features, which can be satisfactorily explained by the MR—MR—MME model of recombination. First, although a typical NF-κB binding site consists of only 10 base pairs, a sequence of 22 bp is copied faithfully. The 22 bp duplicated sequence (5′-GCTGACACAGAAGGGACTTTCT-3′) encompasses a five-base partial binding site of the RBEIII motif (5′-GCTGA-3′), followed by an overlapping seven-base binding site for TFII-I (5′-TGACACA-3′), finally followed by the 10 bases of the F-κB binding site (Figure 3A). The 22 bp duplicated sequence is highly conserved without evident sequence variation regardless of the geographical and temporal origin of the viral strains. Second, the F-κB-motif, comprising the 10 base pairs (italicized) at the 3′ end of the 22 base sequence duplicated, is genetically distinct from the canonical H-κB motif (5′-GGGACTTTCC-3′). The F-κB motif contains a C-to-T base substitution at position 10 of the element (5′-GGGACTTTCT-3′), a molecular trait highly consistent without exception. Thus, a recombination model must satisfactorily explain how the 22 bases of the motif cluster sequence are duplicated along with a mechanism that accounts for the ‘C-to-T’ variation at position 10 of the κB-motif.

The MHD-dependent recombination and mismatched-base extension (MR—MR—MME) model posits that a series of successive processes lead to sequence motif duplication: the RT stalling at the MHDs triplet, the non-specific addition of an ‘A’ residue to the growing end of the cDNA by the extendase activity of the RT, the RT strand-switching to the MHDa motif on the donor RNA template, and a potential mismatch extension by the RT [33]. Considering the sequences with and without the 22-base duplication, we suggest that the RT pauses at the 5′ ‘G’ residue of the GCU motif serving as the MHDs, following the reverse transcription of the H-κB motif, the TFII-I motif, and a partial RBEIII core motif (Figure 3B). The paused RT adds a nucleotide to the growing end of the DNA in the RNA:DNA hybrid in a template-independent manner, typically with a strong preference for an ‘A’ residue [35]. Subsequently, the nascent DNA and donor RNA hybrid dissociates, and the nascent DNA anneals with the acceptor RNA template. When the nascent DNA hybridizes with a cognate complementary sequence on the acceptor RNA, only homologous recombination occurs without sequence duplication. A new RT molecule recognizes the resulting ‘nascent DNA-acceptor RNA’ complex and resumes polymerization that should not introduce variation as the non-specifically added ‘A’ can pair with the natural ‘U’ present on the acceptor template RNA at this position. Alternatively, and rarely, the nascent DNA may misalign with the acceptor RNA template at the MHDa ‘GCU’ triplet, serving as the acceptor for hybridization (Figure 3B). However, this hybridization causes the misalignment of non-complementary bases (C vs. A) at the growing end of the cDNA. The RT then proceeds to extend the mismatched base at the growing end of the nascent DNA, efficiently resuming polymerization, resulting in the ‘C to T’ transition of the F-κB element at position 10 of the motif.

In summary, the duplication of the 22-base sequence motif in HIV-1C depends not only on where the RT pauses in the viral modulator region but also on the RT’s ability to non-specifically add an ‘A’ residue to the growing end of the nascent DNA molecule using the extendase activity and to extend the mismatched base pair at the growing end of the cDNA while resuming polymerization [35].

A second pathway to the creation of the F-κB motif may also exist, where the RT stalls at the 5′ U, immediately upstream of the MHDs GCU sequence. In this case, the RT would add the corresponding ‘A’ nucleotide on the cDNA before transitioning to MHDa on the acceptor strand, bypassing the addition of the non-templated ‘A’. The subsequent steps of misalignment on the acceptor RNA and extension of the mismatched bases would be identical to the previous pathway described above. Additional examples of sequence motif duplications that can be classified as MR—MME duplications are enlisted (Figure 1).

### 2.3. The NR—ME Duplication

An examination of several other events of sequence duplications in HIV-1C did not reveal the presence of a triplet that could serve as an MHD. Nevertheless, we identified a single base within the triplet at the growing end of the cDNA being complementary to the acceptor RNA sequence. Of note, several studies previously illustrated recombination events mediated by single-base pair complementarity [28,29,36,37]. Thus, depending on whether the single base at the terminal position of the cDNA serves as a complementarity residue, the NR model of recombination may be classified into two categories. We provide here examples of some of these recombination events from the duplication hotspots of HIV-1C.

**Figure 4:**
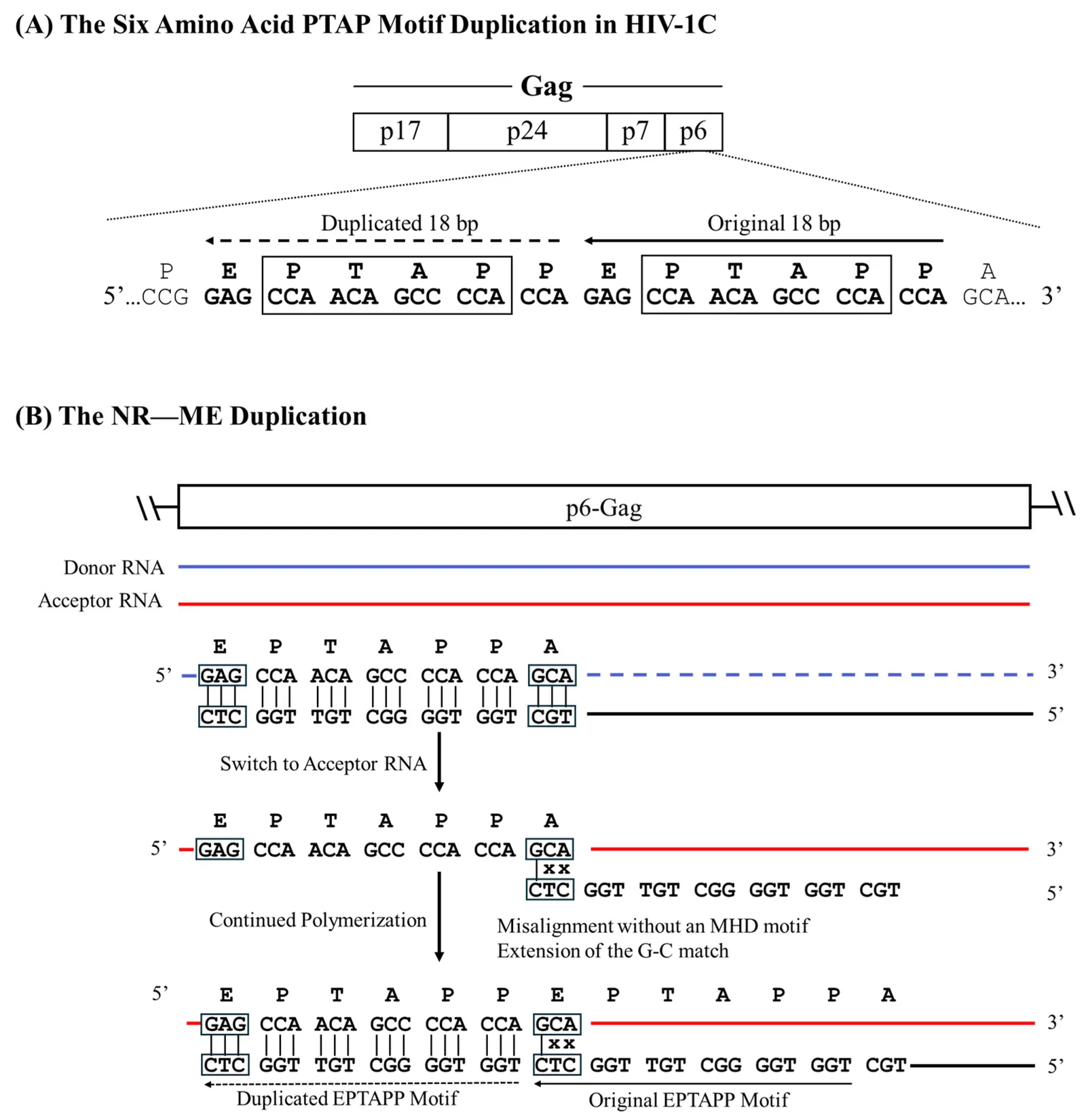
A schematic portraying six-amino-acid PTAP motif duplication in HIV-1C by the NR—ME mechanism. (**A**) Two copies of the PTAP motif of six-amino-acid length (EPTAPP) and the corresponding nucleic acid sequence are presented. Solid and dashed arrows represent the original and the duplicated sequences, respectively. The 18-base sequence in p6-Gag corresponds to 2145–2166 coordinates in HXB2. (**B**) The donor and acceptor RNAs are shown using blue and red lines, respectively, and the black line represents the nascent cDNA. The triplets highlighted by square boxes show the alleged MHD that do not exist in this strategy, and the mismatched bases are represented with an x.

The six amino-acid long PTAP motif (EPTAPP) duplication in HIV-1C p6-Gag serves as a notable example of the NR—NE recombination model. As mentioned previously, six-amino-acid PTAP motif duplication is the second most common duplication in the gag sequences of HIV-1C (23.7%) and HIV-1B (21.7%) available in extant databases. Here, the RT is expected to stall at the 5′ G residue of the ‘GAG’ codon, encoding the Aspartic Acid residue of the ‘EPTAPP’ motif on the donor RNA. Subsequently, the RT switches to the acceptor RNA and mis-pairs with the ‘GCA’ codon encoding the c-terminal Alanine residue of the ‘EPTAPP’ motif on the donor RNA molecule. The pairing adjacent to the ‘CCA’ codon of the cDNA and the ‘GCA’ codon of the acceptor RNA molecule leads to a perfect match at the terminal base located at the growing end, which is recognized by an RT molecule to initiate polymerization. Thus, despite the absence of an evident triplet MHD motif, the misaligned sequence allows the terminal ‘C’ of the cDNA to pair with the ‘G’ of the ‘GCA’ codon, resulting in non-MHD-dependent recombination and matched-base extension (NR—ME) duplication (Figure 4). Additional examples of sequence motif duplications of this kind are listed in Figure 1.

### 2.4. The NR—MME Duplication

Previously, we reported the presence of several LTR-variant viral strains of HIV-1 in India [16]. The example of LRhR-HHC LTR, an LTR variant, serves aptly to explain the NR—MME sequence duplication; additional examples are listed (Figure 1). This duplication creates an additional copy of the RBEIII binding site (R) and the κB-like motif (‘h’, 5′-GGGACTTTCA-3′, deviates from the canonical ‘H-κB motif at position 10, difference underlined). HHC represents two H-κB and one C-κB motifs in the enhancer, and L represents the TCF-1α/LEF-1 binding site. The generation of the LRhR-HHC variant entails the duplication of a 26 bp sequence (5′-AGACTGCTGACACAGAAGGGACTTTC-3′) accommodating binding sites for three TF families—RBEIII (bolded), TFII-I (underlined), and the κB-like element (italicized).

**Figure 5:**
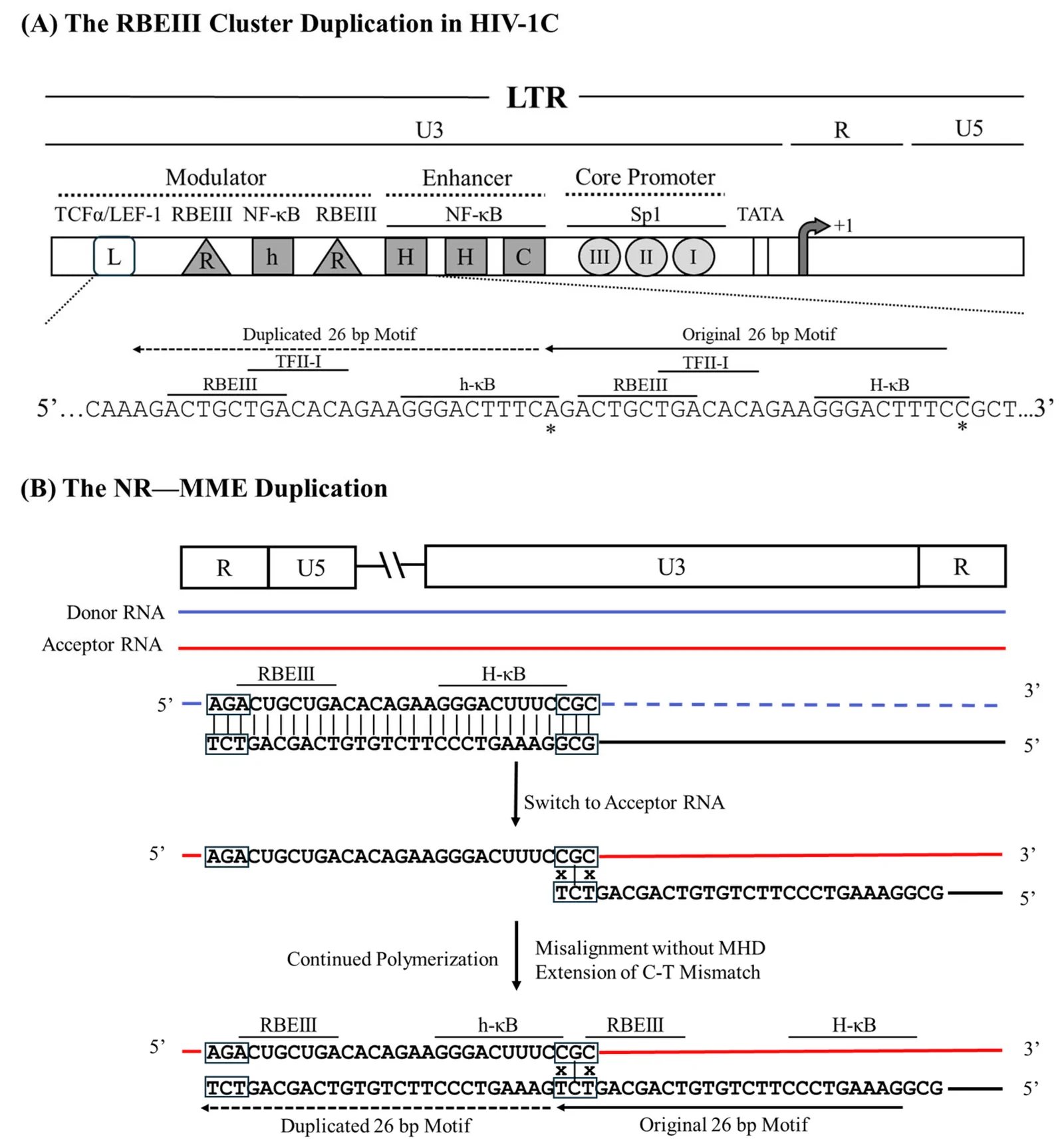
A schematic demonstrating 26 bp RBEIII cluster duplication in HIV-1C by the NR—MME model. (**A**) The organization of the HIV-1C LTR presents RBEIII cluster duplication, the important TFBSs, and their corresponding nucleic acid sequences. Solid and dashed arrows represent the original and the duplicated sequences, respectively. The asterisks represent the C-to-A variation between the original and duplicated motifs. The 26-base pair sequence in the LTR corresponds to 320–352 coordinates in HXB2. (**B**) The donor and acceptor RNAs are shown using blue and red lines, respectively, and the black line represents the nascent cDNA. The absence of the MHD is highlighted using black boxes, and the mismatched bases are represented with an x.

Analysis of the variant LTR sequence fails to find a triplet MHD motif flanking the duplicated sequence to serve as a catalyst to promote a misaligned recombination event. Sequence analysis suggests that the RT pauses at the ‘A’ residue immediately upstream of the RBEIII motif on the donor RNA template (Figure 5). Subsequently, the RT switches the template strands and aligns with the ‘CGC’ triplet on the acceptor RNA immediately downstream of the 5′ H-κB site, thus creating a base pair mismatch between the growing end of the cDNA and the acceptor RNA. The RT commences reverse transcription, disregarding the ‘C-to-T’ mismatch at the growing end of the cDNA, leading to the duplication of the 26 bp sequence and fixing of the ‘C-to-A’ variation at position 10 of the newly formed NF-κB motif and resulting in non-MHD-dependent recombination and mismatched-base extension (NR—MME).

## 3. A Model Elucidating the Structural Constraints That RT Encounters While Duplicating Sequence Motifs

The RT encounters two or three structural and mechanistic challenges while attempting to accomplish the task of duplicating a sequence motif. Misalignment of the cDNA growing end to MHDa causes the formation of a single-stranded DNA loop structure, which should be accommodated within the confines of the RT interior during the process of polymerization. The additional DNA loop occupying the limited space within the active zone of the RT may create mechanistic barriers that may prevent the polymerization process.

Here, we propose a schematic model integrating the fundamental principles of retroviral recombination with insights gleaned from the literature and our own theoretical analysis to elucidate how the RT overcomes these structural constraints to complete the process of polymerization successfully, leading to the creation of a duplicated sequence.

**Figure 6:**
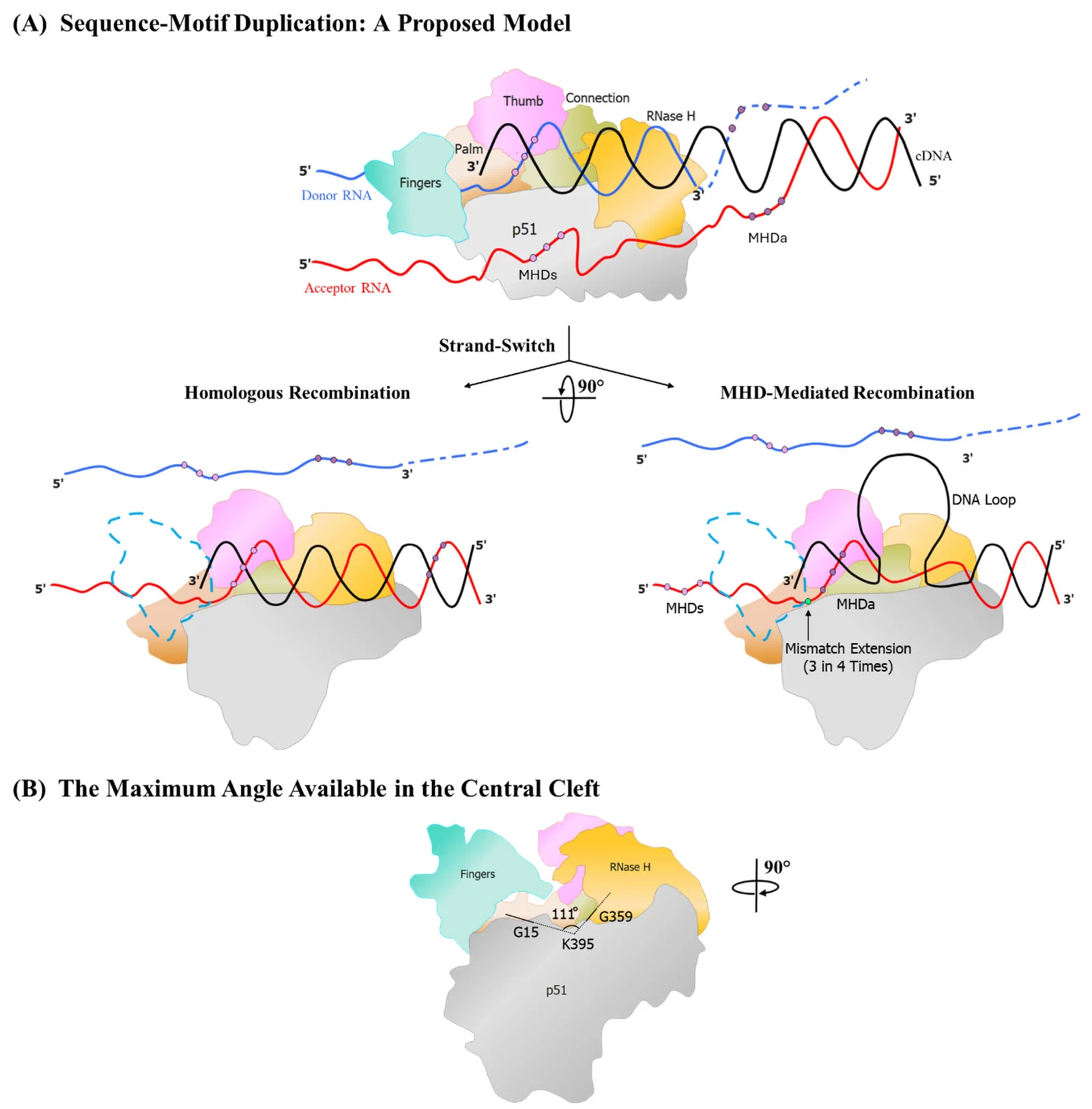
A generalized model for sequence duplication. The model portrays the duplication of an approximately 22-bp sequence motif. The blue and red horizontal lines represent the two template RNA molecules, donor and acceptor, respectively, and the black line the nascent cDNA being polymerized by the RT. The dashed lines represent template RNA hydrolyzed by the RNase H activity of the RT. Three dots in dark and light purple color represent the triplet micro-homology domains MHDa and MHDs, respectively. The terminal base that RT extends during sequence duplication is highlighted in green. Different RT domains are depicted in different colors, as shown. (**A**) The depiction of the RT stalling at the MHD and the acceptor RNA invasion from the 3′ end of the nascent DNA. The two possible outcomes at this event are shown using bifurcated arrows. For clarity, the RT complex is rotated 90° from the top panel. To aid the visualization of the polymerase active site in this view, the finger domain is rendered transparent, with the blue dotted lines depicting its outline. The first outcome of the strand-switch, resulting in homologous recombination, is shown in the central left panel, where the alignment of the cDNA and the acceptor RNA template is perfect. The central right panel displays the misalignment of the cDNA at MHDs, leading to loop formation. The base on the template that is paired incorrectly is shown using a green dot on the DNA. (**B**) The maximum angle available for the rotation of the RT–nucleic acid hybrids in the central space of the RT is depicted after rotating the complex a further 90° from the central panel, as shown. This view represents the RT as it is seen by the cDNA when it enters the cleft. The direction of polymerization is perpendicular to the page surface. The available angle is measured across the G15, K 395, and G359 residues, as shown. Dashed lines are used to display the sections of the angle that are obstructed by the domains of the RT.

To depict this model, we will illustrate the earlier example of 22-base-pair sequence duplication in the LTR using the MR—MME mechanism, leading to the creation of an additional NF-κB motif, the F-κB motif (Figure 3). During minus strand synthesis, the RT, after pausing at the ‘GCU’ MHDs triplet, adds an ‘A’ residue to the growing end of the cDNA through a template-independent process (Figure 3A and Figure 6). At this time, 24 base pairs of the RNA-DNA hybrid are enclosed within the RT [38,39].

The downstream sequence of the donor RNA, having served as the template for reverse transcription, is hydrolyzed by the RNase H activity of the RT. Consistent with the dock-and-lock model, the complementary single-stranded DNA hybridizes with the acceptor RNA molecule in this region of the viral genome. Consequently, the nascent DNA is hybridized with both the donor and the acceptor RNA molecules in different regions, thus generating a trimolecular complex (Figure 6A). Subsequently, when the RT dissociates from the donor RNA, the cDNA aligns with the cognate complementary sequence on the acceptor RNA molecule, and the polymerization process resumes efficiently.

Alternatively, in rare cases, when the RT dissociates from this trimolecular complex, the free bases on the growing end of the cDNA form an intramolecular secondary structure, such as a loop, causing the cDNA to misalign with the MHDa triplet on the acceptor RNA molecule. The secondary structure on the cDNA could be a contributory factor to the RT favoring such a misalignment. Importantly, the formation of a looped secondary structure of the intervening bases of the cDNA represents an inevitable outcome, since these bases lack a complementary region to hybridize with.

**Figure 7:**
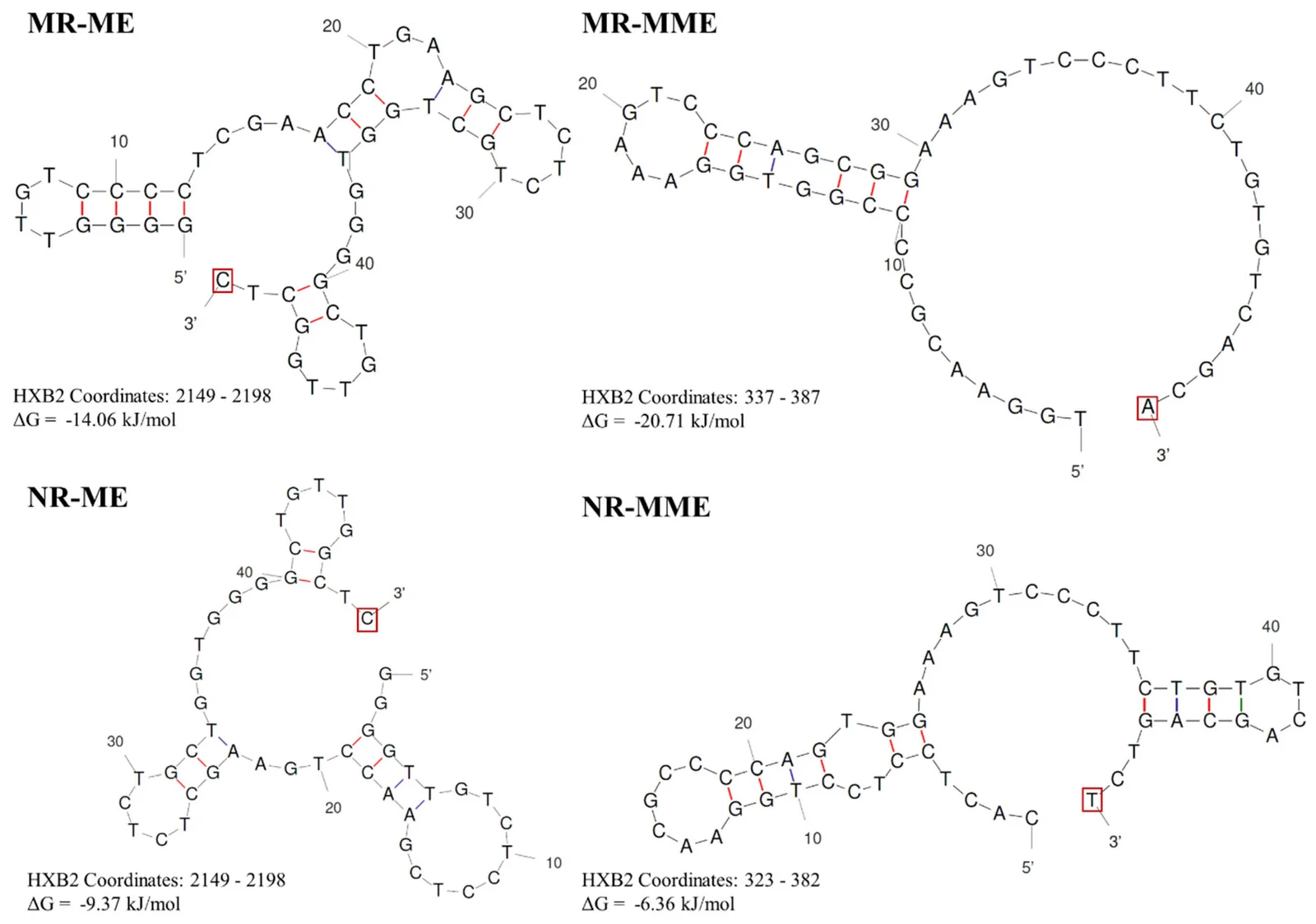
DNA secondary structures in four types of sequence duplications. A representative minus strand cDNA sequence from each of the four proposed sequence duplication categories was used as input for the Unafold DNA secondary structure prediction software available at http://www.unafold.org/ (accessed on 9 February 2024). The conditions for the structure prediction software were set at physiological concentrations of 14 mM and 0.5 mM for Na+ and Mg++ ions, respectively. The predictions characterized by the least ΔG values are presented. The terminal base at the 3′ end that the RT must extend in each case is highlighted using a red square box.

The unhybridized sequences, therefore, must extend outward from the central DNA binding groove of the RT (Figure 6A). The intervening DNA sequences forming such a loop structure could be as long as 48 base pairs, based on sequence evidence available from the extant databases. The RT then extends the cDNA despite the base pair mismatch at the growing end due to the propensity of the HIV-1 RT to read through such mismatches [40,41,42,43,44] (Figure 3B and Figure 6B). The presence of an MHD at the 3′ end of the cDNA facilitates this process and ensures that the reinitiation of polymerization is easier. However, while the MHD is important for the RT to resume polymerization, the formation of the loop is not dependent on the presence of an MHD, since the NR—ME and NR—MME duplications lack a defined MHD. In such cases, the loop is most stabilized only by internal hydrogen bonding (Figure 7), and the RT extends the 3′ terminus despite the absence of significant homology [40,41,42,43,44].

Importantly, the DNA loop protruding from the RT complex would be a significant impediment when polymerization resumes. Analyzing the crystal structure of HIV-1B RT archived in the Protein Data Bank (PDB ID: 5J2M), we determined that the width of the DNA binding groove ranges from 16.3 Å, at its narrowest point between the R78 and L289 residues of the p66 chain, to 46.1 Å, at its widest between the G15 and D471 residues of the p51 and p66 chains, respectively (Figure 6B). The space available within the confines of the RT central cleft is adequate for the DNA loop to extend outward, away from the RT. Notably, during polymerization, each time a fresh base is added to the growing end of the cDNA, the RT must undergo a rotation of approximately 20–30° relative to the axis of polymerization [45]. The DNA loop also must rotate concomitantly along with the RT as a fresh base is added to the cDNA. However, due to the orientation of the DNA loop within the groove, a free unhindered rotation is permitted only up to 111° (as measured across the G359, K395, and G15 residues). Therefore, the RT can add a maximum of three to five nucleotides before the DNA loop encounters the p51 subunit, which impedes further reverse transcription. At this juncture, the RT complex must disassemble, freeing the DNA-RNA complex, and polymerization must resume with a different RT molecule, reassembling the polymerization complex. The process of the addition of up to five nucleotides, complex dissociation, and reassembly must repeat for several rounds until the DNA loop resolves and exits the RT complex, allowing the RT to resume normal polymerization. Our model predicts that incorporating the initial 3–6 base pairs poses the greatest challenge for the enzyme due to the proximity of the loop to the active site. As the loop moves away from the active site, the rate of polymerization accelerates until the process is normalized. This prediction aligns with the observation from the databases that the error rate is highest in bases within three to six positions from the junction between the original and duplicated sequences in the absence of an MHD (Panchapakesan et al., unpublished data).

Our model incorporates the widely accepted tenets of retroviral recombination, representing the dynamic copy choice model and the strand invasion theory to explain how sequence duplications are generated. Importantly, building on the earlier proposition that the cDNA must loop to generate a sequence duplication [28], our model posits that this looped DNA secondary structure is the central driving force underlying a sequence duplication. The formation of this secondary structure is supported by DNA secondary structure predictions using the Unafold Webserver with the 50 terminal bases of representative sequences from all four types of sequence duplications described above. Importantly, in all the secondary structure predictions shown, the terminal base is free for extension by the RT (Figure 7). Further, our model proposes that the events leading up to the RT switching between the two templates are identical between homologous and non-homologous recombination processes. The difference lies in the nonavailability of the looped bases for hybridization with the acceptor RNA template. Therefore, the cDNA is forced to misalign and duplicate the bases copied already.

While the focus of this manuscript is on sequence duplication, the same principles could be extrapolated to sequence deletion. Typically, sequence deletions are far more frequent than sequence motif duplications [46,47], although both events share a common mechanism. For a sequence deletion to occur, the looped secondary structure must form on the template RNA, unlike in sequence duplication, where the cDNA forms the secondary structure. While sequence duplications typically result from inter-molecular switching of the RT enforced by the degradation of the donor RNA template by the RNase H activity of the enzyme, in contrast, deletions can occur due to both inter- and intra-molecular switching of RT [28]. Furthermore, RNA secondary structures are far more numerous than those of DNA, partially explaining the significantly higher proportion of deletions compared to duplications [47].

## 4. Sequence Motif Duplications Are Subjected to Darwinian Selection

Nearly 95–98% of all HIV-1 proviruses present in the latent reservoir are impaired by sequence deletions, frameshift mutations, G-to-A hypermutations, and other defects [46,48,49]. Therefore, substantial efforts have been dedicated to assessing the impact of such genetic defects on latent reservoir properties. In contrast, sequence duplication remains a relatively unexplored area in HIV-1 research, even though sequence duplications of variable length dot the viral genome in many viral strains. However, such sequence duplications are sporadic and lack evident evolutionary patterns. The nature of sequence motif duplications described here in two hotspots, the LTR and p6-Gag, of the viral genome, especially in HIV-1C, deviate by conferring a significant replication fitness advantage on the variant viral strains.

Non-homologous recombination between two template RNA molecules is a precondition for the emergence of sequence motif duplication. However, the frequency of non-homologous recombination is expected to be much lower than that of homologous recombination. For instance, the RT typically switches strands three to five times per replication round, with a recombination probability estimated at 0.03–0.05% per base. Non-homologous recombination, which is responsible for duplication, occurs 100 to 1000 times less frequently than homologous recombination [27]. Since only a fraction of these events result in duplications, the mean duplication rate at a specific genome location is approximately 0.000015–0.0025%.

Importantly, although the frequency of sequence motif duplication is extremely low, the variant viral strains are subjected to Darwinian selection upon their generation. Since sequence duplications are a result of sequence misalignment, two out of every three sequence duplication events are expected to lead to frameshift mutations, resulting in their negative selection. Further, we have previously reported multiple sequence duplications with a high mutation rate at the junction where the RT resumes polymerization on the acceptor RNA template [14,15,16]. The mutations in these duplications, classified as NR—ME and NR—MME in the present work, may also impact viral fitness, affecting their selection.

On the other hand, certain duplications, especially in the hotspots that we have characterized in the LTR and the Gag regions [14,15,16,17,18,19], are associated with a replication advantage, which aids in their positive selection, despite the rarity of their generation [27]. Two factors contribute to this phenomenon. Firstly, the high replication rate of HIV-1 produces approximately 10^10^ to 10^12^ new virions [50] daily in an infected individual, partially compensating for the infrequent appearance of sequence duplications, despite the less defined numbers of infected cells and reverse transcription events. Secondly, certain variant strains containing motif duplications exhibit a replication advantage over wild-type strains, experiencing strong positive selection. We have previously shown that in competitions between viral variants discordant for PTAP duplication, double-PTAP variants consistently outperformed single-PTAP strains in various conditions, including natural infection [14]. As a result, intense positive selection ensures the continued propagation of these variants despite their low frequencies. The domination of these variant viral strains over their canonical counterparts may also influence their transmission potential. In summary, the delicate balance between the rarity of sequence duplications and subsequent positive selection ensures the replication fitness of these variant viral strains.

### Sequence Motif Duplications Represent an Important Evolutionary Mechanism

Sequence duplications that create an additional copy of a biologically significant motif can confer a replication fitness advantage on variant viral strains through quantitative and/or qualitative gains of function. For instance, the viral enhancer of HIV-1B, which contains two tandemly arranged and genetically identical copies of the canonical H-κB motif (5′-GGGACTTTCC-3′), enhances the transcriptional strength of the viral promoter by recruiting the same host factor complexes, such as the p50–p65 heteroduplex, at both sites. In contrast, the canonical enhancer of HIV-1C, which harbors three tandem copies of the NF-κB motif that genetically represent two variable binding sites (two copies of the H-κB motif and one copy of the C-κB motif, 5′-GGGGCGTTCC-3′, differences underlined), may benefit from an additional advantage of recruiting diverse NF-κB dimers to the enhancer. Similarly, the creation of the fourth NF-κB motif in the HIV-1C enhancer by the duplication of 22 bases (Figure 3) introduces additional genetic diversity and proportionately increases transcriptional strength [15]. Thus, viruses like HIV-1 appear to prefer the duplication of short-length sequences, given the packaging restrictions. Consequently, viral strains harboring additional copies of motifs of biological significance are positively selected [14,15,16].

The duplication of shorter and biologically important motifs appears to play a crucial evolutionary role in viruses. The addition of short-length sequences does not significantly alter the genome size, thus avoiding packaging problems. While the original copy of the motif ensures the functional integrity of the biological property, the acquired and genetically variable copies of the motif may benefit variant viral strains by broadening their biological function [14,15,16,17,18]. These variations, whether in open reading frames or regulatory elements, are subjected to strong selection processes.

Taken collectively, the non-homologous recombination models presented here can explain the various observations associated with sequence duplications in HIV-1C. These include genetic variations within the duplicated regions, the high mutational rate at the junctions, the presence or absence of the MHD in certain sequences, and the relatively higher frequencies of some duplications over others. Furthermore, while a few published reports [28,51] have predicted the formation of a secondary structure, we propose that the cDNA secondary structure’s role in hindering correct base pairing with the acceptor RNA molecule serves as the primary driving force for sequence motif duplication. Empirical validation of the recombination models proposed here could prove challenging, given the extremely low frequencies of non-homologous recombination.

## Data Availability

The original contributions presented in this study are included in the article. Further inquiries can be directed to the corresponding author.

## List of Abbreviations

HIV	Human Immunodeficiency Virus
RT	Reverse Transcriptase
APOBEC	Apolipoprotein B mRNA-editing catalytic polypeptide-like
LTR	Long Terminal Repeat
TAR	Trans-Activation Response
TFBS	Transcription factor binding site
MHD	Micro-homology domain
MR—ME	MHD-dependent recombination and matched-base extension
MR—MME	MHD-dependent recombination and mismatched-base extension
NR—ME	Non-MHD-dependent recombination and matched-base extension
NR—MME	Non-MHD-dependent recombination and mismatched-base extension
